# Digital documentation of treatment data and their relation to breed and performance parameters of sows

**DOI:** 10.5194/aab-67-431-2024

**Published:** 2024-09-16

**Authors:** Hannah Görge, Imme Dittrich, Thore Wilder, Nicole Kemper, Joachim Krieter

**Affiliations:** 1 Institute of Animal Breeding and Husbandry, Christian-Albrechts University, Olshausenstraße 40, 24098 Kiel, Germany; 2 Institute of Animal Hygiene, Animal Welfare and Farm Animal Behaviour, University of Veterinary Medicine Hannover, Foundation, Bischofsholer Damm 15, 30173 Hanover, Germany

## Abstract

The emerging risk of resistance to antimicrobials has brought about the need to document animal production in the European Union. The required documentation of treatment data on-farm may withhold viable information, which can be analysed if made accessible in a digital format. The aim of the study was to use a digital system for the documentation of treatments and to investigate relationships between treatments, performance parameters and breeds. Data were collected between August 2020 and September 2022 on a combined pig farm in northern Germany. A digital tool was used to obtain data on treatments, while sows' performance information and breed data were drawn from the sow planner of the farm. Treatment indices were generated for the sows (TIS, treatment index sow) and the sows' litters (treatment index litter, TIL). During the time of data collection, 17 % ( n = 113) of the farrowing events ( n = 661) took place with farrowing-related treatments (FRTs), and 43.5 % ( n = 94) of the sows had to receive FRT at least once. Linear mixed models were used to investigate the dependent variables TIS, TIL and FRT and the performance parameters piglets born alive, stillborn piglets and weaned piglets. The explanatory variables parity, season, year, the interaction of season and year, and the breed of the sow (crossbred Large White × Landrace and purebred Large White), as well as the random effect of the sow nested in the breed of the sow, were included in the models for all dependent variables. Additionally, the breed of the boar (Duroc, Pietrain), TIS, TIL and FRT were investigated in the models of the performance parameters. The following effects were all significant ( p<0.05 ). Parity had an effect on TIS, piglets born alive and stillborn piglets. Sows with parity of 1 had the highest least-squares mean (LSM) = 0.42 (standard error (SE) = 0.04) for TIS, which decreased over parities of 2–3, 4–5 and 6–7 (LSM = 0.16, SE = 0.04) and increased for parity of ≥8 (LSM = 0.34, SE = 0.05). While sows with parity of 1 produced the lowest numbers and sows with parities of 4–5 the highest numbers of piglets born alive, the number of stillborn piglets increased with parity number, with the lowest numbers for parity of 1 and the highest numbers for parity of ≥8 . Overall, the performance of the farm improved in the second year of data collection with less FRT, more weaned piglets and fewer stillborn piglets. Crossbred sows produced more piglets born alive and weaned piglets but also more stillborn piglets. The use of Pietrain boars led to more piglets being born alive. FRT and high treatment indices of the sow related with an increase in stillborn piglets and high treatment indices of the sow resulted in fewer piglets born alive. In conclusion, digitalized treatment data allow for analyses of the herd's health status, and relations between production performance and treatments can be investigated.

## 1 Introduction

The European Union approached the growing risk of resistance to antimicrobials with the Animal Health Strategy in 2007, the EU One Health Action Plan Against Antimicrobial Resistance in 2017 and Regulation (EU) 2019/6 on veterinary medicines alongside Regulation (EU) 2019/4 on medicated feed (More, 2020; European Communities, 2007; European Commission 2017; European Parliament and the Council of the European Union, 2018a, b). Next to setting a basis for coordinated data collection on the sale and use of antimicrobial medicinal products Europe-wide, Regulation (EU) 2019/6 also sets uniform standards for the on-farm documentation of treatment data carried out by the farmer.

At the same time, an era of digitalization has begun, where precision livestock farming (PLF) and the internet of things (IoT) have gained popularity in animal productions to ease workload and continuously monitor animal parameters for welfare, health and production performance assessments (Morrone et al., 2022). The potential of modern technologies to ease workload is of interest as the farming industry develops towards increased farm sizes and experiences difficulties finding and maintaining permanent workers. At the same time, consumers demand high standards of welfare in animal production, and with the help of PLF (e.g. sensors), early-warning systems can help the farmer maintain these high welfare standards (Buller et al., 2020; Halachmi et al., 2019; Alonso et al., 2020). In terms of treatment documentation, in contrast to handwritten documentation, digitalized data are easily accessible for analyses and can be used to interpret events of the past, to identify issues in the present and to predict the future (Eastwood et al., 2019; Janssen et al., 2017; Wolfert et al., 2017). Thus, knowledge of treatment prevalence and occurrence patterns of diseases can be gained and used for disease prevention strategies.

In the piglet production industry, efficiency is essential for economic viability. Two important cost components are veterinary costs and replacements, while profit is dependent on sows' production performance (Krieter, 2002). A sow's health status influences its economic value (Rodríguez et al., 2011). This influence can be traced back to the direct negative effects of diseases on production performance such as litter size, stillborn piglets or weaned piglets or indirect negative effects due to early removal of the sows (Anil et al., 2009, 2005b; Niemi et al., 2017; Pluym et al., 2013; Andersson et al., 2020; Bardehle et al., 2012). Especially lameness has been associated with early removals of sows, and farrowing-related diseases such as postpartum dysgalactia syndrome (PDS) are associated with negative effects on production performance (Anil et al., 2005b, 2009; Bardehle et al., 2012). PDS is a multifactorial disease complex associated with agalactia, mastitis, metritis and unspecific symptoms of the sows such as fever, inappetence and apathy, which does not only affect the sow but also the piglets as they are dependent on sufficient milk supply (Maes et al., 2019). Therefore, health status should be considered next to factors such as parity, season and breed in the evaluation of sows' reproductive performance.

The available information could also be beneficial for selection purposes, as heritabilities have been associated with health parameters. For PDS, heritabilities ranging from 2 % to 20 % have been estimated in different studies (Preissler et al., 2012; Krieter and Presuhn, 2009; Lingaas and Rønningen, 1991; Berg et al., 2001). Even though the heritabilities are low, selection for healthy animals can lead to the breeding of robust genetics, following the example of Heringstad et al. (2003) regarding mastitis in cows. Additionally, breeds of pigs have been shown to differ in their susceptibility to certain diseases such as *Porcine reproductive and respiratory syndrome virus* (PRRSV) and *Porcine circovirus type 2* (Opriessnig et al., 2006; Petry et al., 2005). Thus, treatment information may be able to pinpoint breeds in need of more or less medical support.

Görge et al. (2023) compared existing systems for the documentation of treatment data on-farm, which differed in their ability to document within the scope of European law and within the application process and to document treatments linked to the individual animals. While most systems lacked external validations, and the reliability of on-farm documentations was criticized, the value of said systems was highlighted.

The aim of the study was to investigate relationships of sow treatments, especially farrowing-related diseases and treatments of the sows' litters, to performance parameters. Therefore, a digital tool for documentation of treatment data was used. It can fulfil the requirements of the law and trace the treatments to the individual.

## 2 Material and methods

### 2.1 Animals and housing

Data collection took place at a combined pig farm located in Schleswig-Holstein, Germany. The pig farm operated as a closed system and replaced sows with self-produced gilts. The pigs' housing followed regulations according to the EU Council Directive 2008/120/EC, EU Council Directive 2010/63/EC, and the 2017 “German Order for the Protection of Production Animals used for Farming Purposes and other Animals kept for the Production of Animal Products” (TierSchNutztV). At the time, the farm kept a mean number of 147 productive sows. Integrated in the study were n = 170 crossbred Large White × Landrace sows (hereafter referred to as crossbred), n = 46 purebred Large White sows (hereafter referred to as purebred) and n = 17 sows of other breeds (Duroc and crossbred Large White × (Large White × Landrace)). Production performance parameters are displayed in Table 1. The sows were artificially inseminated with the genetics of Duroc ( n = 297, 1 to 13 times the same boar) and Pietrain ( n = 249, 1 to 18 times the same boar) for piglet production purposes and with the genetics of Large White and Landrace for breeding purposes ( n = 38). In n = 15 cases, the semen of the house boar was used, and in n = 6 cases, mixed semen was used. The pig farm functioned as a test station for an artificial insemination organization (GFS, Ascheberg, Germany); hence semen from various boars was tested. All sows were equipped with radio frequency identification (RFID) ear tags.

**Table 1:** Overview of the production performance of the sows in the study represented by mean ( $\bar{x}$ ) and standard deviation (SD). All: all sows on the farm during the study period. Purebred is Large White and crossbred Large White × Landrace.

Production performance parameter	All		Purebred		Crossbred	
	$\bar{x}$	SD	$\bar{x}$	SD	$\bar{x}$	SD
Litters per sow per year	02.3 ${}^{*}$	–	–	–	–	–
Piglets weaned per sow per year	31.3 ${}^{*}$	–	–	–	–	–
Piglets born alive per litter	15.1	4.5	14.1	4.5	15.4	4.4
Stillborn piglets per litter	01.2	1.7	0.8	1.2	1.2	1.7
Piglets weaned per litter	13.2	3.5	12.5	3.7	13.4	3.2
Parity	04.4	2.6	4.8	3.2	4.2	2.4
Parity of sows that remained in the group	03.9	2.4	–	–	–	–
Parity of sows that left the group	05.3	3.0	–	–	–	–

${}^{*}$ Number drawn directly from the farm's sow planner.

At the beginning of the production cycle, the sows were kept 28 to 36 d individually in crates at the insemination unit of the farm, with slatted concrete flooring in the back and solid concrete flooring in the front of the crate. Here, they were fed two portions of dry freed at 07:00 and at 07:30 CET (central European time), respectively, and the amount of feeding varied depending on the sow's condition. All crates were equipped with bowl drinkers. The temperature of the insemination unit was kept at 20 to 21 °C. Afterwards, the sows were moved into group housing, with a maximum group size of 60 sows. The lying areas were equipped with concrete flooring, while the activity areas had slatted concrete flooring. Gilts were kept in two groups, separated from the other sows, with 13 sows each. Both group housing systems were equipped with on-call feeders, where dry feed was provided to each sow individually depending on the state of gestation. Water was provided with bowl drinkers as well as nipple drinkers. The temperature in the group housing systems was kept at 19 to 20 °C. A week before the calculated farrowing date, the sows were washed and moved to the farrowing compartments. The sows were fixed in farrowing crates in four of the five farrowing compartments. These compartments contained 10 to 12 pens each with slatted plastic flooring. The fifth farrowing compartment consisted of three pens. Here, the sows were put into the crates only 1 d before the calculated date of farrowing and were released 5 d after farrowing. The plastic flooring was slatted despite solid flooring along a small area on the wall-facing side of the crate and the separated piglet nest. The sows were fed twice daily at 07:00 and at 15:30 until the sixth day of lactation. Afterwards, they were fed three times daily with an additional feeding time at 10:30. Water was provided with bowl drinkers for the sows and nipple drinkers for the piglets. The farm operated a 3-week farrowing cycle and a suckling period of 28 d. On the first day of life, every piglet received a RFID ear tag, which was removed after identification at slaughter because the pig farm tested the offspring for the artificial insemination organization. Litters were homogenized in the first days after farrowing. The piglets were kept with undocked tails. In the first week of the piglets' lives, the male piglets were castrated surgically under injection anaesthesia. Next to the sow's milk, the piglets were offered dry feed. At the beginning of the suckling period, the temperature in the farrowing departments was kept at 21 °C and was then reduced to 20 °C over the 28 d period. After weaning, the sows were moved to the insemination unit to start the next production cycle. The piglets stayed in the farrowing pens for 3 more days and were then moved to the rearing stable. The rearing stable was separated from the piglet production and partly equipped with slatted plastic flooring. Here, the piglets were kept in groups of 40 to 45 individuals and fed with moist feeding ad libitum with a ratio of 3 : 1 animal to feeding place. When reaching an age of 63 to 67 d, the piglets were transferred to the fattening stable, which was also separated. The stable consisted of 10 compartments, of which 8 had 12 pens and 2 had 4 pens each. All pens had slatted concrete flooring, and 15 fattening pigs were kept in each pen. Since April 2022, only 13 fattening pigs have been kept in each of the pens. The fattening pigs were fed restrictively three times a day at 05:00, 10:30 and 14:30 with liquid feed at a ratio of 1 : 1 animal to feeding place. The pigs stayed in the fattening stable for 3–4 months until slaughter.

### 2.2 Treatment routine

Prophylactic measures in terms of vaccinations and anthelmintic treatments were carried out routinely. Gilts received vaccinations against *Actinobacillus pleuropneumoniae, Erysipelas, Influenza, Mycoplasma hyopneumoniae, Parvovirus* and *Porcine circovirus*. Lactating sows received vaccinations against *Erysipelas* and *Parvovirus.* Gestating sows received vaccinations against *Clostridia, Escherichia coli, Erysipelas* and *Parvovirus*. The piglets were vaccinated against *Lawsonia intracellularis*, *Mycoplasma hyopneumoniae* and *Porcine circovirus*. The anthelmintic treatments were carried out for the sows when they were moved into the farrowing pen and for the fattening pigs when they were moved into the fattening stable. In the farrowing compartments, mammary glands, potential discharge originating from the parturient organs, the sows' appetite and the sows' behaviour were examined. On the first 2 d after farrowing, the rectal temperature of each sow was measured; temperatures above 39.4 °C were considered a fever. Daily animal observations were carried out in all stables to identify pigs showing symptoms of illness, for example, coughs, lameness or tail lesions. Treatments were administered to ill pigs as advised by the supervising veterinarian.

### 2.3 Data collection

The digital collection of treatment data at the time of administration was carried out with the V-ETIC system (Henke Sass Wolf GmbH, Tuttlingen, Germany, last version number used: 3.2.0) from August 2020 to September 2022. The V-ETIC system is cloud-based and connects to a mobile application. The mobile application connects to an RFID reader via Bluetooth, which is attached to a self-filling syringe. At the time of treatment administration, the animal's RFID ear tag was identified, and the treatment data, previously selected from a drop-down list within the mobile application, linked to the individual were saved in the mobile application that was also to be used offline. For synchronization with the cloud, a wi-fi connection was needed. The treatments were provided as treatment lists in the cloud. For the purpose of digital treatment documentation, a mobile phone and a RFID reader were placed at each of the production areas. No changes in the treatment routine were made due to the system implementation. From August 2020 to January 2022, weekly farm visits were carried out to provide support for system use and check for completeness of documentation. The digitally documented treatment data were compared to the handwritten documentation, which was carried out simultaneously and completed when, for example, treatments were missing. From February to September 2022, the treatment data were extracted from the cloud-based system only, as the weekly farm visits were discontinued. Group treatments were not documented as the link to the individual was not given, and the linkage of the individuals RFID to the group treatments was not feasible to be integrated into the farm's procedures.

Additionally, data were drawn from lists that were created throughout the whole period of data collection on the first day of the piglets' lives by the farm staff using another digital device (WORKABOUT PRO™ 3, Psion Teklogix Inc., Mississauga, Canada), depositing the link of the sow's RFID to the piglet's RFID, birthdate and weight with the help of an integrated RFID reader. Performance data of each farrowing cycle, including parity of the sow, piglets born alive, stillborn piglets and weaned piglets, and heritage data of the sows and piglets were obtained from the farm's sow planner AGROCOM SUPERSAU (CLAAS, Harsewinkel, Germany). Lastly, data on removal of sows of the herd and reasons for this were obtained from the farms' documentation in the form of Excel tables.

### 2.4 Data restriction and treatment indices

System malfunctions led to loss of treatment data in December 2020 in the rearing stable, April and May 2021 in all production areas, and between 27 May and 2 June 2022 in the piglet production and rearing stable. Of the 729 farrowing events in August and September 2020, 30 had to be excluded (4 % exclusion) in order to provide a balanced dataset for the integration of the comparison of the seasons. For the investigation of the breed of the sow, 38 further farrowing events were excluded (9 % total exclusion) in order to compare only crossbred sows to purebred sows. This results in a dataset containing 661 farrowing events of 216 sows used for the most analyses. To only compare the Duroc and Pietrain breeds for boars, 148 further farrowing events were excluded (29 % total exclusion), resulting in 513 farrowing events of 199 sows used for these analyses. Technical issues occurred with the device linking the piglets' RFID to the sow on the first day of the piglets' lives, resulting in missing values for 52 of the 661 farrowing events included in the big dataset, resulting in 609 (18 % total exclusion) farrowing events of 211 sows used for these analyses.

Overall, there were 1151 treatments documented digitally for the sows. After exclusion of duplicate treatments (0.3 % exclusion), prophylaxis treatments (e.g. vaccinations, 7.8 % exclusion), treatments of unknown indication (6.7 % exclusion), treatments not traceable to individuals (1.5 % exclusion) and addition of treatments using handwritten documentation ( n = 40), 1003 observations of treatments remained. The treatments were categorized according to their indication in farrowing-related treatments, treatments of the locomotor system and other treatments. In order to determine whether treatments were farrowing-related, they were compared in relation to the farrowing date. Taking other studies considering farrowing-related diseases in sows and the farm's procedures into account, any treatments started within 5 d prior to and 5 d after farrowing were counted as farrowing-related treatments. When system malfunctions occurred, manual treatment documentation was used to fill the gap in documentation. With this information, each farrowing event of a sow was documented regarding whether a farrowing-related treatment occurred (FRT = 1) or not (FRT = 0).

A treatment index of the sow was created in accordance with the antibiotic treatment index of QS Qualität and Sicherheit GmbH (Bonn, Germany) (QS), in which the treatment index of the sow equals the sum of treatment units within one farrowing cycle (QS Qualität und Sicherheit GmbH, 2023). A treatment unit was defined as the number of treatment days multiplied by the number of active ingredients. A farrowing cycle was defined for sows of parity 1 from the date of the integration into the herd to first weaning and for all other parities from weaning to weaning. As QS and the German antibiotic monitoring scheme use similar practices to differentiate farms and production areas with the most antibiotic usage from the other farms, a distribution analysis of the treatment index was conducted, and a threshold was drawn at the third quartile to differentiate from the other sows the sows which needed the most treatments. Sows with a treatment index above the third quartile were assigned a treatment index sow (TIS) value of 1, and the ones within the third quartile were assigned a TIS value of 0 (Federal Office of Consumer Protection and Food Safety, 2005).

A total of 5551 observations of treatments were documented for the piglets in the piglet production, the rearing stable and the pigs in the fattening stable. Only 4562 observations of individual treatments remained after exclusion of duplicate treatments, treatments of unknown indication and treatments without linkage to individuals. Taking the lists generated on the first day of the piglets' lives into account, the treatments were assigned to the farrowing events of the sows. With this information, a treatment index of each sow's litter was created, also following the treatment index of QS:

$$\mathrm{treatment}\;\mathrm{index}\;\mathrm{litter}=\frac{\mathrm{sum}\;\mathrm{of}\;\mathrm{treatment}\;\mathrm{units}\;\mathrm{per}\;\mathrm{litter}}{\mathrm{number}\;\mathrm{of}\;\mathrm{piglets}\;\mathrm{in}\;\mathrm{the}\;\mathrm{litter}}.$$

Here, a treatment unit was defined as the number of individuals treated multiplied by the number of treatment days and the number of active ingredients. Following the approach of the aforementioned treatment index of the sow, a distribution analysis of the treatment index of the litter was conducted to identify litters needing the most medical attention. The threshold was set at the third quartile again, assigning the litters above the third quartile with a treatment index litter (TIL) value of 1 and the litters within the third quartile a TIL value of 0.

### 2.5 Statistical analyses

All analyses were carried out using the statistical software SAS^®^ 9.4 (SAS Institute Inc., 2018).

#### 2.5.1 Investigation of treatments

Linear mixed models (MIXED procedure) were built to analyse potential effects of the generated treatment indices and farrowing-related treatments in particular. The binary variables TIS, FRT and TIL served as the dependent variables in three separate models. Usually, binomial distributions do not allow for the use of mixed models. Still, the de Moivre–Laplace theorem implies that in large sample sizes, binomial distributions follow nearly a normal distribution, and the following applies:

$$B_{n;p}(k)\approx \frac{1}{\sigma }\cdot \phi \left(\frac{k-\mu }{\sigma }\right)=\frac{1}{\sqrt{2\pi \sigma 2}}\cdot e^{-\frac{1}{2}\cdot {\left(\frac{k-\mu }{\sigma }\right)}^{2}},$$

with the expected value μ=n⋅p and the variance $\sigma^{2}=n\cdot p\cdot (1-p)=n\cdot p\cdot q$ , with n being the sample size (e.g. observations), p the probability of success and q=1-p . A normal distribution can be assumed if n⋅p⋅q≥9 applies. For all three binary variables, the statistical requirements were fulfilled. The variables' parity (1, 2–3, 4–5, 6–7, ≥8 ), season (summer is April to September, and winter is October to March), year (first year is October 2020 to September 2021, and second year is October 2021 to September 2022), season ⋅ year and breed of the sow (crossbred, purebred) were integrated into the models in a stepwise manner considering Akaike's information criterion (AIC) to evaluate the models' goodness of fit. Additionally, the random effect of the sow nested in the breed of the sow was included in all three models.

#### 2.5.2 Investigation of performance parameters

Linear mixed models were also built for the analyses of the effects of the treatment indices and farrowing-related treatments on performance parameters. The response variables piglets born alive, stillborn piglets and weaned piglets were investigated. In a first step, parity, season, season ⋅ year and the breed of the sow were integrated in a stepwise manner as explanatory variables, building the basis models for the performance parameters. In a second step, the variables TIS (0,1), FRT (0,1), TIL (0,1) and the breed of the boar (Duroc, Pietrain) were integrated separately, resulting in four models for each performance parameter investigated. In the process of model building, the breed of the boar was integrated both as a single variable in addition to the basic model and in addition to the variables TIS, FRT or TIL. Due to the reduction of the observation number associated with the variable breed of the boar, the final models included the breed of the boar as a single variable in addition to the basic model. Lastly, the sow nested in the breed of the sow was included as a random effect in all models. To evaluate the goodness of fit, the AIC was considered.

For interpretation of the models, the level of significance was set at p≤0.05 , and to interpret the data, the least-squares mean (LSM), the standard error (SE) and differences, with their level of significance adjusted by Bonferroni, were considered.

## 3 Results

### 3.1 Investigation of treatments

From 661 farrowing events integrated into the statistical models, 113 (17 %) farrowing events took place with farrowing-related treatments (FRTs). Out of 216 sows, 94 sows received FRT at least once, which represents 43.5 %. Of the 94 sows, 77 (81.9 %) received farrowing-related treatments at one farrowing event, 15 (16.0 %) at two and 2 (2.13 %) at three events. Sows were integrated with up to five farrowing events.

The LSM and SE of the three models explained in this section are presented in Table 2. In the model regarding TIS, parity ( p=0.01 ) had a significant effect. Parity of 1 had the highest LSM = 0.42 (SE = 0.04). The LSM declined over parities of 2–3 and 4–5, and parities of 6–7 had the lowest LSM = 0.16 (SE = 0.04) and increased again for parity of >8 with LSM = 0.34 (SE = 0.05). Parities of 6–7 differed significantly from parities of 1, 2–3 and >8 . In the model regarding FRT, only the variable year ( p=0.04 ) had a significant effect. There were more FRTs in the first year (LSM = 0.23, SE = 0.03) in comparison to the second year (LSM = 0.16, SE = 0.03). The interaction of season and year ( p<0.01 ) had a significant effect on TIL. Values started with LSM = 0.09 (SE = 0.04) in the first winter, LSM = 0.33 (SE = 0.04) in the first summer, LSM = 0.45 (SE = 0.04) in the second winter and LSM = 0.07 (SE = 0.04) in the second summer. The repeatability varied between the parameters from 0.0 % to 2.0 % (TIS = 1.0 %, FRT = 2.0 % and TIL = 0.0 %) in the models described.

**Table 2:** Least-squares mean (LSM) and standard error (SE) for farrowing-related treatments (FRTs), treatment index sow (TIS) and treatment index litter (TIL). n is the number of farrowing events. Bold letters indicate statistical significance ( p<0.05 ). A–C indicate significant differences.

Explanatory variables		FRT		TIS		TIL	
		n = 661		n = 661		n = 609	
		LSM	SE	LSM	SE	LSM	SE
Parity	1	0.26	0.04	**0.42** ${}^{A}$	**0.04**	0.24	0.04
	2–3	0.19	0.03	**0.33** ${}^{A}$	**0.04**	0.22	0.04
	4–5	0.18	0.04	**0.28** ${}^{\mathrm{AB}}$	**0.04**	0.24	0.04
	6–7	0.15	0.04	**0.16** ${}^{B}$	**0.04**	0.31	0.04
	>8	0.21	0.04	**0.34** ${}^{A}$	**0.05**	0.16	0.05
Season ${}^{1}$	Summer	0.20	0.03	0.30	0.03	**0.20**	**0.03**
	Winter	0.20	0.03	0.31	0.03	**0.27**	**0.03**
Year ${}^{2}$	First	**0.23**	**0.03**	0.31	0.03	0.21	0.03
	Second	**0.16**	**0.03**	0.30	0.03	0.26	0.03
Season ⋅ year	First winter	0.23	0.03	0.35	0.04	**0.09** ${}^{A}$	**0.04**
	First summer	0.23	0.03	0.28	0.04	**0.33** ${}^{B}$	**0.04**
	Second winter	0.17	0.03	0.28	0.04	**0.45** ${}^{C}$	**0.04**
	Second summer	0.17	0.03	0.31	0.04	**0.07** ${}^{A}$	**0.04**
Breed sow ${}^{3}$	Purebred	0.23	0.04	0.35	0.05	0.23	0.05
	Crossbred	0.17	0.02	0.26	0.02	0.24	0.02

${}^{1}$ Summer is April to September, and winter is October to March. ${}^{2}$ First year is October 2020 to September 2021, and second year is October 2021 to September 2022. ${}^{3}$ Purebred is Large White and crossbred Large White × Landrace.

### 3.2 Investigation of performance parameters

The LSM and SE of the models including TIS, FRT and TIL explained in this section are presented in Table 3.

**Table 3:** Least-squares mean (LSM) and standard error (SE) for the performance parameters piglets born alive, weaned piglet and stillborn piglets. n is the number of farrowing events. FRT is the farrowing-related treatment, TIS is the treatment index sow, TIL is the treatment index litter. Bold letters indicate statistical significance ( p<0.05 ). A–C indicate significant differences.

Explanatory		Piglets born alive								Weaned piglets								Stillborn piglets							
variables		n = 661		n = 661		n = 609		n = 513		n = 661		n = 661		n = 609		n = 513		n = 661		n = 661		n = 609		n = 513	
		LSM	SE	LSM	SE	LSM	SE	LSM	SE	LSM	SE	LSM	SE	LSM	SE	LSM	SE	LSM	SE	LSM	SE	LSM	SE	LSM	SE
Parity	1	**12.6** ${}^{A}$	**0.4**	**12.6** ${}^{A}$	**0.4**	**12.9** ${}^{A}$	**0.4**	**12.5** ${}^{A}$	**0.5**	13.3	0.3	13.4	0.3	13.6	0.3	13.4	0.4	**0.5** ${}^{A}$	**0.2**	**0.4** ${}^{A}$	**0.2**	**0.3** ${}^{A}$	**0.2**	**0.3** ${}^{A}$	**0.2**
	2–3	**13.8** ${}^{A}$	**0.4**	**13.6** ${}^{A}$	**0.4**	**14.3** ${}^{A}$	**0.4**	**13.9** ${}^{\mathrm{AB}}$	**0.5**	12.3	0.3	12.4	0.3	12.9	0.3	12.3	0.4	**0.7** ${}^{A}$	**0.2**	**0.6** ${}^{A}$	**0.2**	**0.5** ${}^{A}$	**0.2**	**0.6** ${}^{A}$	**0.2**
	4–5	**16.2** ${}^{B}$	**0.4**	**16.1** ${}^{B}$	**0.4**	**16.4** ${}^{B}$	**0.4**	**16.3** ${}^{C}$	**0.5**	12.9	0.3	13.0	0.3	13.1	0.3	13.0	0.4	**1.1** ${}^{B}$	**0.2**	**1.0** ${}^{B}$	**0.2**	**0.9** ${}^{A}$	**0.2**	**0.9** ${}^{A}$	**0.2**
	6–7	**15.3** ${}^{B}$	**0.5**	**15.1** ${}^{B}$	**0.4**	**16.0** ${}^{B}$	**0.4**	**15.9** ${}^{C}$	**0.5**	12.5	0.3	12.5	0.3	13.1	0.3	13.0	0.4	**1.8** ${}^{C}$	**0.2**	**1.7** ${}^{C}$	**0.2**	**1.5** ${}^{B}$	**0.2**	**1.6** ${}^{B}$	**0.2**
	>8	**15.5** ${}^{B}$	**0.5**	**15.4** ${}^{B}$	**0.2**	**15.8** ${}^{B}$	**0.5**	**15.1** ${}^{\mathrm{BC}}$	**0.6**	12.6	0.4	12.7	0.4	13.0	0.3	12.6	0.5	**2.2** ${}^{C}$	**0.2**	**2.1** ${}^{C}$	**0.2**	**2.0** ${}^{B}$	**0.2**	**2.0** ${}^{B}$	**0.2**
Season ${}^{1}$	Summer	14.4	0.3	14.3	0.3	14.6	0.3	**14.3**	**0.4**	12.9	0.2	12.9	0.2	13.2	0.2	12.8	0.3	**1.4**	**0.1**	**1.3**	**0.1**	1.1	0.1	1.2	0.2
	Winter	15.0	0.3	14.9	0.3	15.6	0.3	**15.1**	**0.4**	12.6	0.3	12.7	0.2	13.1	0.2	12.9	0.3	**1.2**	**0.1**	**1.0**	**0.1**	1.0	0.1	1.0	0.2
Year ${}^{2}$	First	14.7	0.3	14.6	0.3	15.1	0.3	14.6	0.4	**11.9**	**0.3**	**12.0**	**0.2**	**12.2**	**0.2**	**11.8**	**0.3**	**1.5**	**0.1**	**1.4**	**0.1**	**1.3**	**0.1**	**1.4**	**0.2**
	Second	14.6	0.3	14.5	0.3	15.1	0.3	14.8	0.4	**13.5**	**0.2**	**13.6**	**0.2**	**14.1**	**0.2**	**13.9**	**0.3**	**1.1**	**0.1**	**0.9**	**0.1**	**0.8**	**0.1**	**0.8**	**0.2**
Season ⋅ year	First winter	**15.4** ${}^{A}$	**0.4**	**15.3** ${}^{A}$	**0.4**	**16.0** ${}^{A}$	**0.4**	15.3	0.5	11.8	0.3	11.9	0.3	12.1	0.3	11.9	0.4	1.3	0.2	1.2	0.2	1.1	0.2	1.2	0.2
	First summer	**14.1** ${}^{B}$	**0.4**	**13.9** ${}^{B}$	**0.4**	**14.3** ${}^{B}$	**0.4**	14.0	0.5	12.0	0.3	12.1	0.3	12.3	0.3	11.7	0.4	1.7	0.2	1.6	0.1	1.4	0.2	1.6	0.2
	Second winter	**14.5** ${}^{\mathrm{AB}}$	**0.4**	**14.4** ${}^{\mathrm{AB}}$	**0.4**	**15.2** ${}^{\mathrm{AB}}$	**0.4**	14.9	0.5	13.4	0.3	13.5	0.3	14.0	0.3	13.9	0.4	1.0	0.2	0.9	0.1	0.8	0.2	0.7	0.2
	Second summer	**14.7** ${}^{\mathrm{AB}}$	**0.4**	**14.6** ${}^{\mathrm{AB}}$	**0.4**	**15.0** ${}^{\mathrm{AB}}$	**0.4**	14.7	0.5	13.7	0.3	13.8	0.3	14.2	0.3	13.9	0.4	1.1	0.1	1.0	0.1	0.8	0.2	0.8	0.2
Breed sow ${}^{3}$	Purebred	**14.1**	**0.5**	14.1	0.5	14.9	0.5	14.2	0.7	**12.1**	**0.4**	**12.2**	**0.4**	**12.6**	**0.3**	**12.0**	**0.5**	**1.0**	**0.2**	**0.9**	**0.2**	**0.8**	**0.2**	0.9	0.3
	Crossbred	**15.2**	**0.3**	15.1	0.2	15.3	0.2	15.2	0.2	**13.4**	**0.2**	**13.5**	**0.2**	**13.6**	**0.2**	**13.7**	**0.2**	**1.6**	**0.1**	**1.5**	**0.1**	**1.3**	**0.1**	1.3	0.1
Breed boar	Duroc	–	–	–	–	–	–	**14.3**	**0.4**	–	–	–	–	–	–	12.9	0.3	–	–	–	–	–	–	1.2	0.2
	Pietrain	–	–	–	–	–	–	**15.1**	**0.4**	–	–	–	–	–	–	12.8	0.3	–	–	–	–	–	–	1.0	0.2
FRT	0	14.8	0.3	–	–	–	–	–	–	13.0	0.2	–	–	–	–	–	–	**0.9**	**0.1**	–	–	–	–	–	–
	1	14.6	0.4	–	–	–	–	–	–	12.5	0.3	–	–	–	–	–	–	**1.6**	**0.2**	–	–	–	–	–	–
TIS	0	–	–	**15.0**	**0.3**	–	–	–	–	–	–	13.0	0.2	–	–	–	–	–	–	**0.9**	**0.1**	–	–	–	–
	1	–	–	**14.1**	**0.4**	–	–	–	–	–	–	13.5	0.3	–	–	–	–	–	–	**1.4**	**0.1**	–	–	–	–
TIL	0	–	–	–	–	15.2	0.3	–	–	–	–	–	–	13.1	0.2	–	–	–	–	–	–	1.1	0.1	–	–
	1	–	–	–	–	15.0	0.4	–	–	–	–	–	–	13.2	0.3	–	–	–	–	–	–	1.0	0.2	–	–

${}^{1}$ Summer is April to September, and winter is October to March. ${}^{2}$ First year is October 2020 to September 2021, and second year is October 2021 to September 2022. ${}^{3}$ Purebred is Large White and crossbred Large White × Landrace.

#### 3.2.1 Piglets born alive

For the linear mixed models regarding the piglets born alive, parity ( p<0.01 ) had a significant effect in all four models. Over all four models, parity of 1 had the lowest LSM of piglets born alive and parities of 4–5 the highest LSM of piglets born alive. In the models including FRT, TIS or TIL, parities of 1 and 2 differed significantly from parities of 4–5, 6–7 and >8 . In these models, the interaction of season and year ( p=0.01 ) had a significant effect as well, whereas the first winter and summer differed significantly in their LSM. The first winter had the highest LSM of piglets born alive, and the first summer had the lowest LSM; and in the winter and summer of the second year, the LSM increased again and stabilized. In the model including the breed of the boar, the season ( p=0.03 ) had a significant effect, whereas summer had fewer piglets born alive (LSM = 14.3, SE = 0.4) than winter (LSM = 15.1, SE = 0.4). The breed of the sow ( p=0.05 ) only had a significant effect in the model including FRT. Here, crossbred sows produced more piglets born alive (LSM = 15.2, SE = 0.3) in comparison to purebred sows (LSM = 14.1, SE = 0.5). From the varying explanatory variables, the breed of the boar ( p = 0.03) and TIS ( p = 0.02) had a significant effect. Pietrain breeds (LSM = 15.2, SE = 0.4) produced more piglets born alive than Duroc breeds (LSM = 14.3; SE = 0.4), and sows with TIS = 0 (LSM = 15.0, SE = 0.3) produced more piglets born alive than sows with TIS = 1 (LSM = 14.1, SE = 0.4).

#### 3.2.2 Weaned piglets

For the linear mixed models regarding the weaned piglets, the year ( p<0.01 ) and the breed of the sow ( p<0.01 ) had significant effects on the number of weaned piglets in all four models. In the first year of data collection, there were significantly fewer weaned piglets than in the second year of data collection. Crossbred sows weaned significantly more piglets than purebred sows.

#### 3.2.3 Stillborn piglets

For the linear mixed models regarding the stillborn piglets, parity ( p<0.01 ) and year ( p<0.01 ) were considered to have a significant effect on the number of stillborn piglets in all four models. The number of stillborn piglets increased constantly from parity of 1 to parity of >8 . While parities of 1, 2–3 and 4–5 differed significantly from parities of 6–7 and >8 in all models, parity of 1 differed significantly from parities of 4–5 as well in the models including TIS, FRT and the breed of the boar. In the first year, there were significantly more piglets born alive than in the second year. In the models with FRT or TIS integrated, season ( p=0.03 ) had significant effects on the number of stillborn piglets, with more stillborn piglets in summer than in winter. Additionally, the breed of the sow ( p<0.01 and p = 0.02) had significant effects on the models with FRT, TIS and TIL integrated. Crossbred sows produced significantly more stillborn piglets than purebred sows. From the varying explanatory variables, FRT ( p<0.01 ) and TIS ( p<0.01 ) had significant effects on the number of stillborn piglets. When FRT = 1 (LSM = 1.6, SE = 0.2) there were more stillborn piglets present than when FRT = 0 (LSM = 0.9, SE = 0.1) and analogous for TIS = 1 with LSM = 1.4 (SE = 0.1) and TIS = 0 with LSM = 0.9 (SE = 0.1).

## 4 Discussion

### 4.1 Investigation of treatments

With a prevalence of on average 13 % that is described to reach up to 60 %, PDS is the major farrowing-related disease in pig production (Bäckström et al., 1984; Baer and Bilkei, 2005; Krieter and Presuhn, 2009; Hermansson et al., 1978; Madec and Leon, 1992; Papadopoulos et al., 2010). In this study, FRT includes any farrowing-related treatment with application of pain medication or antimicrobials and not specifically PDS; hence the prevalence of FRT of 17 % is not directly comparable to findings in other studies. PDS is a multifactorial disease complex characterized by reduced milk production that may occur next to mastitis and metritis in the first days after farrowing. Thus, it affects the piglets directly as they are dependent on sufficient colostrum and milk supply to develop an immune system and to nurture (Maes et al., 2010). Furthermore, thresholds for diagnoses and therefore treatments are likely to differ from farm to farm and between different studies. For example, even though fever is one of the most common diagnostic symptoms of PDS, the threshold for fever varies between 39.3 and 40.5 °C (Waldmann and Wendt, 2004). Additionally, farrowing-related diseases may be overdiagnosed in some studies when the main symptom for diagnosis of PDS is fever as hyperthermia in postparturient sows can be physiological (Klopfenstein et al., 2006; Stiehler et al., 2015). Moreover, the definition of time related to the farrowing event in which the disease occurs differs throughout the literature. PDS has been discussed to occur within 12 to 48 h postpartum or even before the event of farrowing, and some studies have considered its occurrence within the first 3–4 d after farrowing (Gerjets and Kemper, 2009; Hoy, 2003; Furniss, 1987; Bertschinger et al., 1990; Baer and Bilkei, 2005; Hirsch et al., 2003). In this study, even treatments starting 5 d past farrowing were taken into account, considering that some treatments administered in the weekend shifts were documented digitally on the following Monday.

Repeated FRTs for the same sows in following farrowing events were present in this study. Hermansson et al. (1978) found that sows with PDS were at greater risk of the syndrome in the following parity, and heritabilities for PDS of 2 % and 20 % have been estimated in the literature (Preissler et al., 2012; Krieter and Presuhn, 2009; Lingaas and Rønningen, 1991; Berg et al., 2001). In this study, the repeatability was at 2 % for FRT, indicating only a small effect of the sow on FRT. Nevertheless, selection on behalf of PDS could be performed with the information available, as Heringstad et al. (2003) showed for mastitis in cows. However, the low heritabilities and multifactorial aetiology of PDS stress the importance of other factors such as hygiene and management optimization (Preissler et al., 2012; Papadopoulos et al., 2010).

There was no significant effect of parity on FRT present but on TIS. As TIS includes all treatments and the main other treatment indication next to FRT is lameness, lameness is emphasized in the following. When new sows are integrated into the group, fights are usually carried out to establish a social hierarchy causing injuries (Anil et al., 2005a). This potentially explains the higher LSM for younger sows for TIS and the decline in LSM as the sows grow older and are more settled in their social structures (Maes et al., 2016). The decrease could also be present due to the removal of diseased sows as lameness increases the risk of removal (Pluym et al., 2013; Anil et al., 2009). The increase in TIS for parity >8 may reflect the finding of Dewey et al. (1993) that sows of higher parities are more likely to have foot lesions.

The TIL was only related to the interaction of season and year that is potentially relatable to the fact that the farm did not vaccinate against PRRSV. Hence, PRRSV infections moving through the piglet production in winter of the first year of data collection could have been the cause for this relation. PRRSV mainly causes respiratory symptoms in piglets but also unspecific symptoms, for example,diarrhoea, nervous-system-related symptoms and rough hair coats. Therefore, high TIL may represent the aftermath of this infection. Furthermore, PRRSV causes late-term reproductive failure and premature farrowing in sows, consequently with high numbers of stillborn piglets (Rossow, 1998; Christianson et al., 1992; Neumann et al., 2005). Hence, it may explain the higher number of stillborn piglets, lower numbers of weaned piglets and higher number of FRT in the first year as well as the decrease in piglets born alive in the first summer of data collection. Neumann et al. (2005) determined a decrease of 22 % in weaned piglets due to PRRSV, analysing PRRSV-affected and PRRSV-unaffected farms.

### 4.2 Investigation of performance parameters

In this study, FRT and TIS were related to the occurrence of more stillborn piglets. This complies with Gerjets et al. (2011) and Bardehle et al. (2012), who also found relations between mastitis or PDS and the number of stillborn piglets. The association of stillborn piglets and FRT may originate in the same risk factors of either. Stillborn piglets were found to be associated with longer duration of farrowing in other studies, which was also associated with fever after farrowing (Tummaruk and Sang-Gassanee, 2013; Oliviero et al., 2010; Canario et al., 2006; Langendijk et al., 2018). Additionally, long farrowing durations increase the risk for uterine inflammation, retained placentas, lower colostrum yield and PDS in general and may cause birth interventions (Björkman et al., 2017; Kaiser et al., 2018; Quesnel, 2011; Weber, 2018). Bardehle et al. (2012) found birth interventions to be a risk factor for contracting PDS. Furthermore, high temperatures in the farrowing unit were found to be associated with stillborn piglets, a reduced feed intake, milk yield and higher body temperature of the sow (Vanderhaeghe et al., 2010a; Quiniou and Noblet, 1999; Messias de Bragança et al., 1998).

In accordance to the results of this study, Anil et al. (2009) found associations between lameness and fewer piglets born alive, and Pluym et al. (2013) found more mummified piglets, which may be reflected in the number of stillborn piglets as they were not differentiated in this study. Nevertheless, Fitzgerald et al. (2012) and Kroneman et al. (1993) did not find associations between lameness and performance parameters. Furthermore, lameness and PDS may increase the risk for removal, which affects the overall production performance of the sow (Pluym et al., 2013; Anil et al., 2009; Hermansson et al., 1978).

The breed of the sow influenced the performance parameters, with more piglets born alive and more weaned piglets for crossbred sows than expected according to the heterosis effect (Sellier, 1976; Johnson, 1981; Smith and King, 1964; Fahmy and Bernard, 1972). Nevertheless, purebred sows had fewer stillborn piglets in this study. Cecchinato et al. (2010) found fewer stillborn piglets in crossbred sows and discussed the variability in different studies to be due to genetic and environmental factors across populations next to differences in the definition of stillborn piglets.

In a study of Pedersen et al. (2019), differences of performance parameters between Duroc and Pietrain were investigated; Pietrain boars were found to be more fertile than Duroc boars, which is in accordance with the present study. However, piglet mortality including the number of stillborn piglets was lower in Duroc boars, which was not found in this study.

As described, stillborn piglets increased with every farrowing, which complies with Leenhouwers et al. (2003) and Randall and Penny (1970). Vanderhaeghe et al. (2010a, b) discussed the risk of stillbirth in sows, showing that higher parities may be due to poor calcium homeostasis or oxytocin secretion in higher parities, which may lead to longer farrowing durations. Roldan-Santiago et al. (2019) investigated the vitality scores of piglets during eutocic farrowings. They found piglets with lower vitality scores including higher levels in partial CO ${}_{2}$ , lower pH levels and higher incidence of meconium staining in the first and seventh parity in comparison to piglets from parities of 2–6. The sows produced the fewest piglets born alive at parity of 1 and the most piglets at parities of 4–5, which is in accordance with Large White, Landrace and Large White × Landrace crossbred sows in the studies of Knecht et al. (2015) and Koketsu et al. (1999). However, higher parities showed constant numbers for piglets born alive.

### 4.3 Digital documentation

In general, a digital system for the documentation of treatments is beneficial to handwritten documentation as double-entry bookkeeping and mistakes made during documentation can be reduced, and the data are available for analyses (Görge et al., 2023). Under European law, it is necessary that the digital documentation is reliable (European Parliament and the Council of the European Union, 2018b). System malfunctions of the V-ETIC system led to data loss and treatments having to be excluded due to missing treatment indication as described above. Looking more closely at the data of missing indications, they were mostly not documented in August 2020, when the system was newly integrated at the farm. This suggests an increased reliability after a certain adaption period of a system on a farm. Furthermore it should not impact the analyses of this study as the first 2 months of farrowing events was excluded, as described in Sect. 2.4. Group treatments could not be documented, linked to the individual animals in this study. For the required documentation, treatment documentations linked to the location are sufficient in the pig industry, but it limits the TIL which should be considered in further studies. As shown and discussed by Moura et al. (2023), there are indications such as gastrointestinal diseases where group treatments are considered to be more efficient and indications such as respiratory diseases which are predominantly treated with oral group treatments (Larsen et al., 2016). In contrast to this study, Sarrazin et al. (2019) only considered group treatments. Nevertheless, it is recommended to give individual parenteral or oral treatments over group treatments in order to prevent antimicrobial resistance (European Medicines Agency, 2019)

## 5 Conclusion

This study highlighted the attention that farrowing-related diseases such as PDS require on an intensive pig-producing farm. Effects of treatments of the sows and their litters were investigated as well as their relation to performance parameters of the sows. Positive relations between stillborn piglets and treatments of the sow in general and farrowing-related treatments in particular were observed. Sows receiving treatments produced fewer piglets born alive. Additionally, the parity of the sow influenced the treatment index of the sow, the occurrence of stillborn piglets and piglets born alive. In conclusion, special care should be taken in the management of gilts and older sows in order to prevent disease and minimize performance loss in the form of fewer piglets born alive and the occurrence of stillborn piglets, in addition to ensuring animal health. Although the technologies did not function completely reliably throughout the study period and group treatments should be considered in future studies, the potential of digital documentation can be deduced from the results of this study.

## Data Availability

Data are available from the corresponding author upon reasonable request.
